#  Sacral Rachipagus Parasite: A Case Report

**Published:** 2016-04-10

**Authors:** Kamal Nain Rattan, Jasbir Singh, Poonam Dalal, Pallavi Sonika, Ananta Rattan

**Affiliations:** 1 Department of Pediatric Surgery, PGIMS, Rohtak, Haryana, India 124001; 2 Department of Pediatrics, PGIMS, Rohtak, Haryana, India 124001

**Keywords:** Conjoined twins, Heteropagus, Parasite

## Abstract

We are reporting a case of sacral rachipagus parasite which was vaginally delivered as a large irregular mass attached to the sacral region by a vascular pedicle. This case was managed successfully by surgical excision of parasite.

## INTRODUCTION

Conjoint twins are rare embryologic developmental anomalies with estimated prevalence of 1 in 50,000 to 1 in 100,000 births [1, 2].They are classified as symmetrical and asymmetrical conjoint twins depending upon maturation of the twins with respect to each other. Parasitic or heteropagus twin are used as synonymous with asymmetric twin. The parasitic twin may be ectoparasite or lie within the fully developed fetus as endoparasite (fetus in fetu) [3]. In asymmetric twining there is an incompletely developed fetus, which is attached to well mature fetus (autosite) at variable sites [4]. In rachipagus, the twins are joined dorsally at vertebral column and they are the rarest type [5]. We report a case of sacral rachipagus parasite.

## CASE REPORT

A 20-years-old primigravida, in labor, was referred with antenatally diagnosed multicystic mass attached to fetus on antenatal ultrasonography. She delivered a 2.6 Kg female baby through vaginal delivery with APGAR score of 7/10 and 9/10 at 1st and 5th minutes of life respectively. There was a large ulcerated sacral mass of size 25cm×20cmwith rudimentary limb attached to baby by a pedicle (Fig. 1a). Serosanguinous discharge was oozing from the mass on superior aspect. A globular mass of size 10cm×10 cm was also delivered along with baby (Fig. 1b). Most probably it was a part of parasite and got detached from it during delivery. In postnatal ultrasonography, there was cystic lesion with foci of calcification in that parasite. As the mass was actively bleeding, emergency surgery was planned after initial stabilization of neonate.

**Figure:**
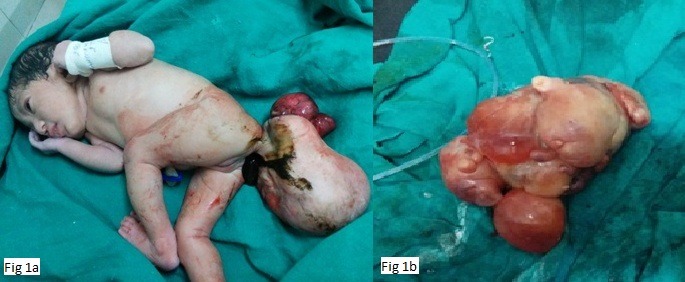
Figure 1: (a) Newborn immediately after delivery with attached sacral parasite (b) Gross morphology of globular mass which was delivered along with the baby

Patient was catheterized and placed in prone position. An elliptical incision was given around pedicle and total excision of parasite mass was done. Feeding blood vessels were ligated and adequate hemostasis was attained. There was no bony connection in between parasite and autosite. The wound was closed in layers without drain.

Postoperative period was uneventful and the patient was discharged on 10th day. During her stay we did x-ray chest, lumbosacral region and an echocardiography which ruled out spina bifida and congenital cardiac anomalies. Histopathological examination showed 3 distinct structures with obvious bony, cartilaginous and neural tissue. Patient is doing well in follow-up with no urinary or stool incontinence.

**Figure:**
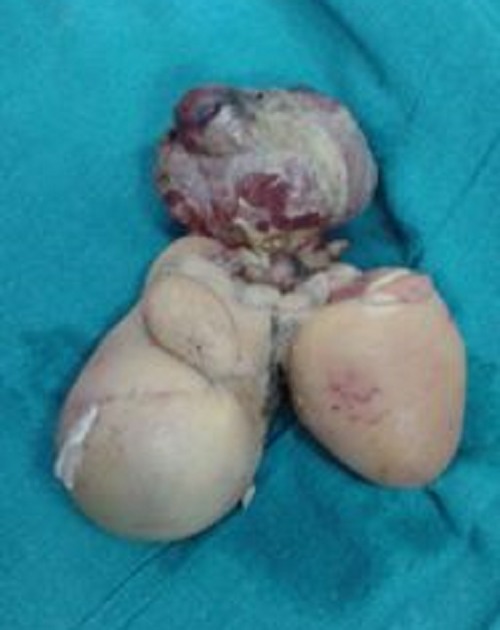
Figure 2: Gross morphology of sacral parasite twin with rudimentary limb

## DISCUSSION

The rachipagus twins are thought to be joined dorsally in the open neural folds before their closure to form neural tube. Parasite twining results from death of one of the twins. Vestigial organs may persist and remain attached to autosite. Although any organ may present but incidence of limb bones are most commonly reported. Variable degree of vertebral column fusion may be seen in rachipagus, with dorsal spine is the most common site of union. In index case parasite was attached with autosite with narrow pedicle and there were no bony connections with the autosite. On extensive search of literature, we fail to find such type of union in sacral parasite. 

Craniovertebral anomalies and neural tube defects in autosite are most commonly associated congenital anomalies with rachipagus parasites [5]. Also when there is extensive fusion of spinal cord, incidence of associated anomalies increases in autosite. These factors are very significant in deciding management plan and outcome of the patient. In the index case there was no bony attachment and no fusion with the spinal cord.

Sacral parasite has to be differentiated from sacrococcygeal teratoma due to malignant potential of later. Parasites usually have development of vertebral axis, variable degree of organogenesis and development of vestigial limbs. But even in absence of vertebral column, some cases are defined as parasites [6]. In index case presence of rudimentary limb, fluid filled sacs, cut section showing bony structures with areas of calcification (Fig. 2) confirmed the diagnosis of parasite twin. Rudimentary limbs may show neuronal innervations but they are not controlled by autosite [7].

Conjoint twins are associated with increased mortality both intra uterine period and after birth. Better antenatal supervision is the most important intervention to reduce the mortality. Antenatal ultrasonography (USG) can pick up the diagnosis as early as 9th week of gestation. On USG, conjoined twins will remain in fixed positions to each other. Early diagnosis is of prime importance so that we can decide whether to continue or terminate pregnancy if associated with life threatening anomalies in autosite. Whenever there is doubt, MRI scan and echocardiography can be used to confirm the diagnosis. In our case antenatal period was not properly supervised. She had done antenatal USG examination only in last trimester of pregnancy at peripheral health centre.

Surgical excision of parasite twin is the treatment of choice [8].Surgical procedures are usually elective but emergency surgery is indicated when there is bleeding from the mass. In present case there was oozing from the parasite due to separation of part of parasite during vaginal delivery hence we had to undertake emergency surgery due to rapidly worsening condition of baby. Single staged surgery was performed in index case. As parasitic twins were associated with lesser degree of organ fusion, they have good outcome after surgery. In conclusion, rachipagus sacral parasite is the rarest variety of heteropagus twining. Prognosis is excellent in absence of life threatening malformations in autosite.

## Footnotes

**Source of Support:** Nil

**Conflict of Interest:** Nil
